# Supplementary material for: Inactivation of Arid1a in the endometrium is associated with endometrioid tumorigenesis through transcriptional reprogramming
Source: Nat Commun. 2020 Jun 1;11:2717. doi: 10.1038/s41467-020-16416-0 (PMC7264300; doi:10.1038/s41467-020-16416-0)

Supplementary Figures for

Inactivation of *Arid1a* in the Endometrium is Associated with Endometrioid Tumorigenesis through Transcriptional Reprogramming

By Suryo Rahmanto et al,

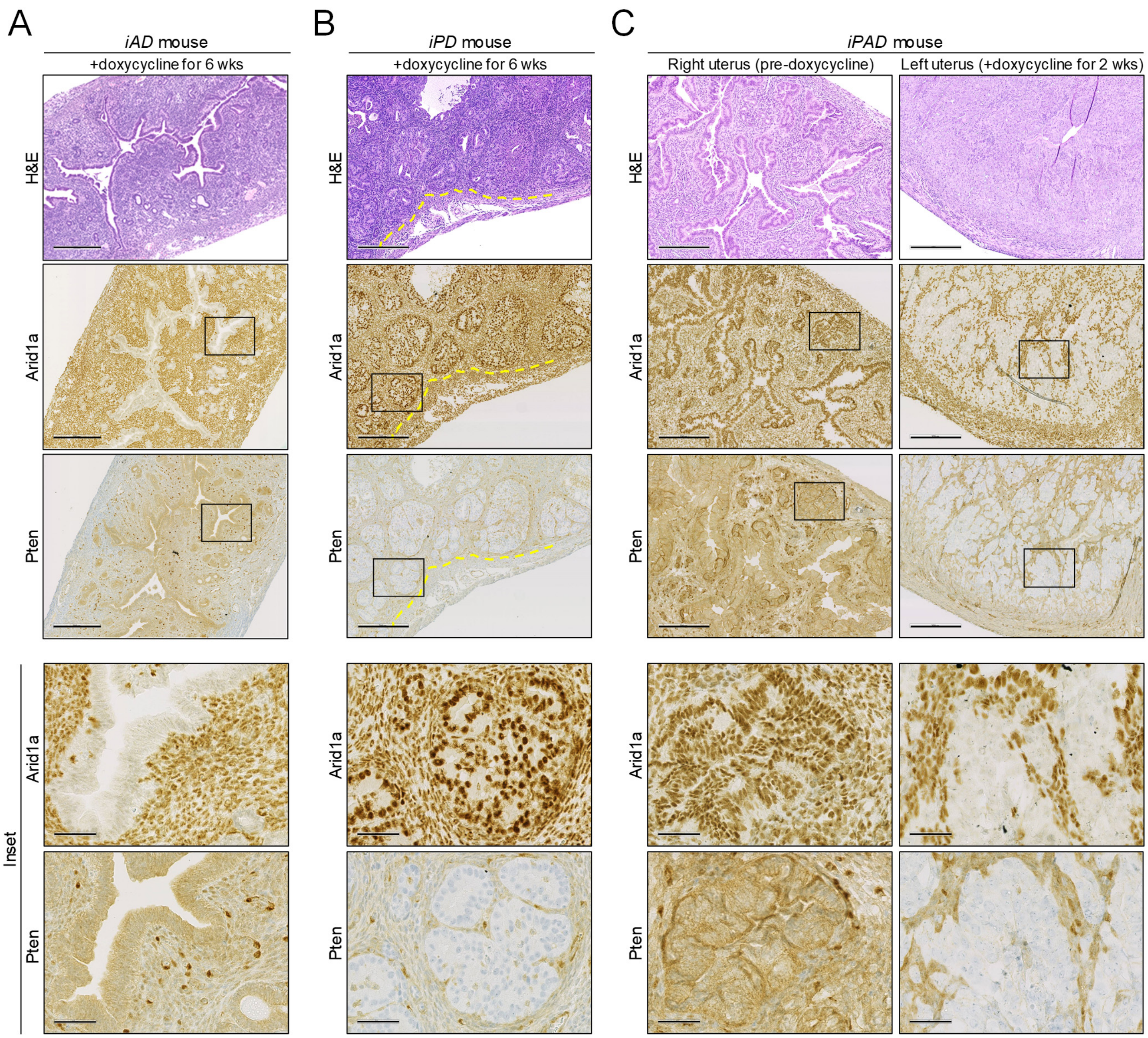

Supplementary Figure 1. Immunohistochemistry and histology performed on uteri of *iAD*, *iPD*, and *iPAD* mice.

(A & B) Arid1a and Pten immunohistochemical staining of uterine tissues from *iAD* and *iPD* mice 6 weeks after doxycycline-induced knockout. No histological abnormality was detected in the endometrium of *iAD* mouse. Atypical hyperplasia with no observable invasion was found in the endometrium of *iPD* mouse. Yellow dashed lines demarcate junctions between hyperplastic gland and normal uterine tissue. Scale bars, 300 µm. **(C)** A representative *iPAD* mouse whose right and left uterine horns were resected at different time point: right uterus obtained before doxycycline treatment and left uterus obtained 2 weeks after doxycycline treatment. Arid1a and Pten immunohistochemical analysis confirms loss expression of both genes. Tumor cells are negative for Arid1a and Pten immunostaining, while stromal cells are positive and serve as the internal positive control. Scale bars, 300 µm. Insets show higher magnification of the indicated area of the respective immunohistochemical stains. Inset scale bars, 50 µm.

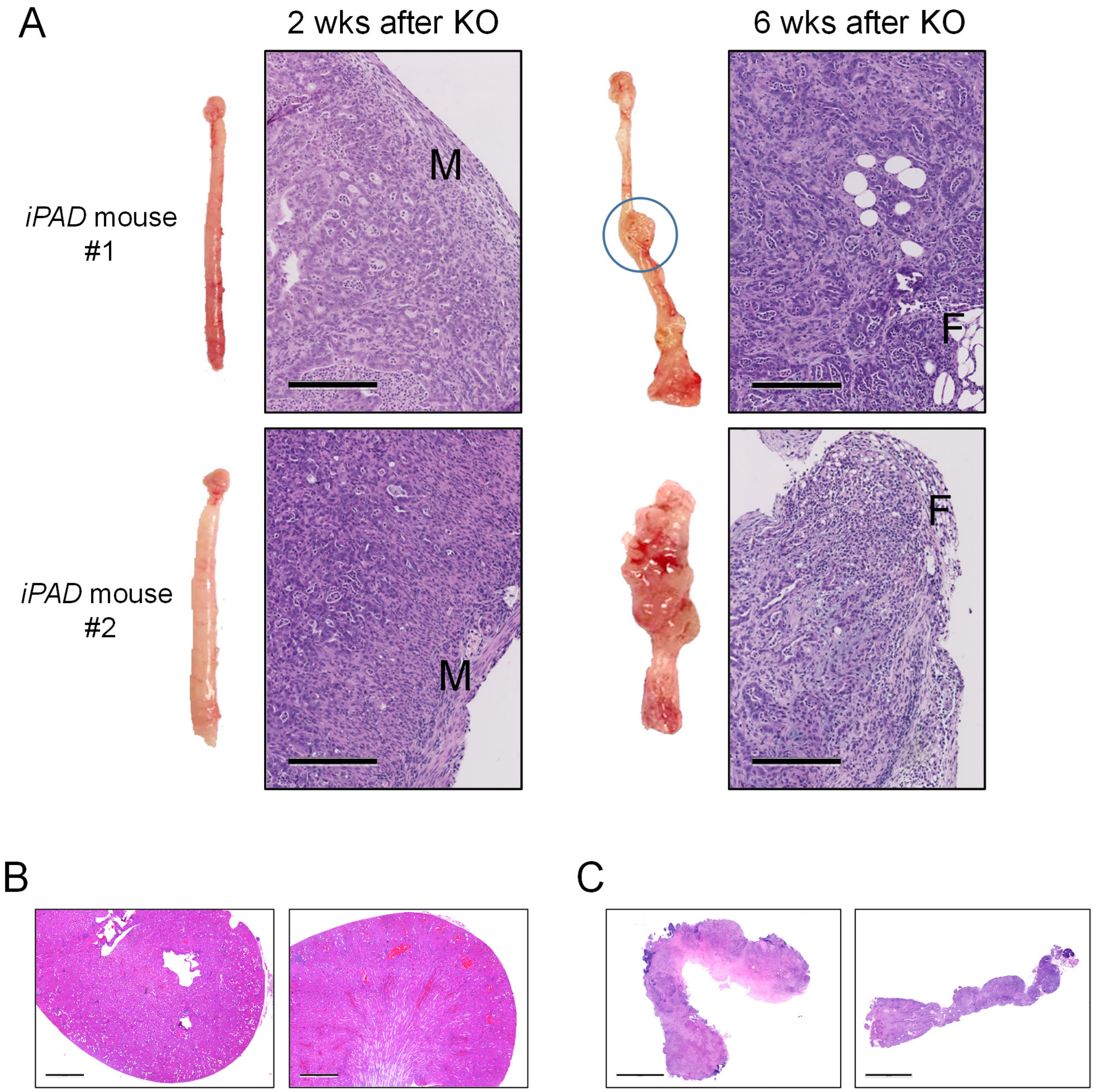

Supplementary Figure 2. Histology of *iPAD* uterine and kidney tissues at different time points after doxycycline-induced gene knockout.
(A) Representative images of bilateral uteri from two *iPAD* mice excised at week 2 (left uterus) and week 6 (right uterus) after doxycycline treatment. At week 2, uteri from both mice do not show gross abnormality but H&E sections reveal endometrial carcinoma without myometrium (M) invasion. At week 6, gross tumors are readily appreciated in the other uteri from both mice. Microscopically, carcinomas invade through the myometrium and infiltrate into the peritoneal fibroadipose tissues (F). Scale bars, 200 μm. **(B)** Representative images of hematoxylin and eosin (H&E) staining of kidney tissues from *iPAD* mice that have been treated with doxycycline for 6 weeks. No gross abnormalities were identified in these *iPAD* derived kidney tissues. Scale bars, 1 mm. **(C)** Representative low magnification images of H&E stained uteri from *iPAD* mice that have been treated with doxycycline for 6 weeks. Scale bars, 5 mm.

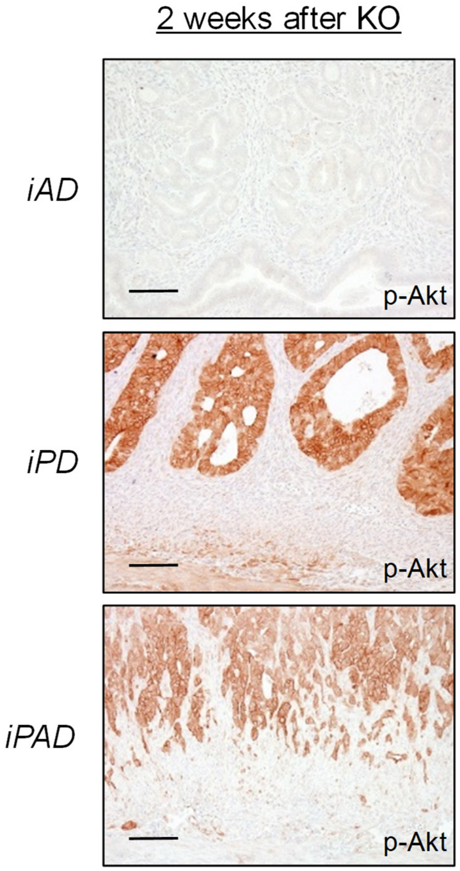

Supplementary Figure 3. Immunohistochemistry of phospho-Akt performed on uterine tissues of *iAD*, *iPD* and *iPAD* mice.
Uterine was excised at 2 weeks after doxycycline-induced gene knockout. Immunohistochemistry was performed on uterine tissues using an anti-p-Akt Ser473 antibody. Immunoreactivity of p-Akt is undetectable at *iAD* uterine mucosa while intense staining is observed in *iPD* and *iPAD* endometrial epithelium. Scale bars, 100 μm

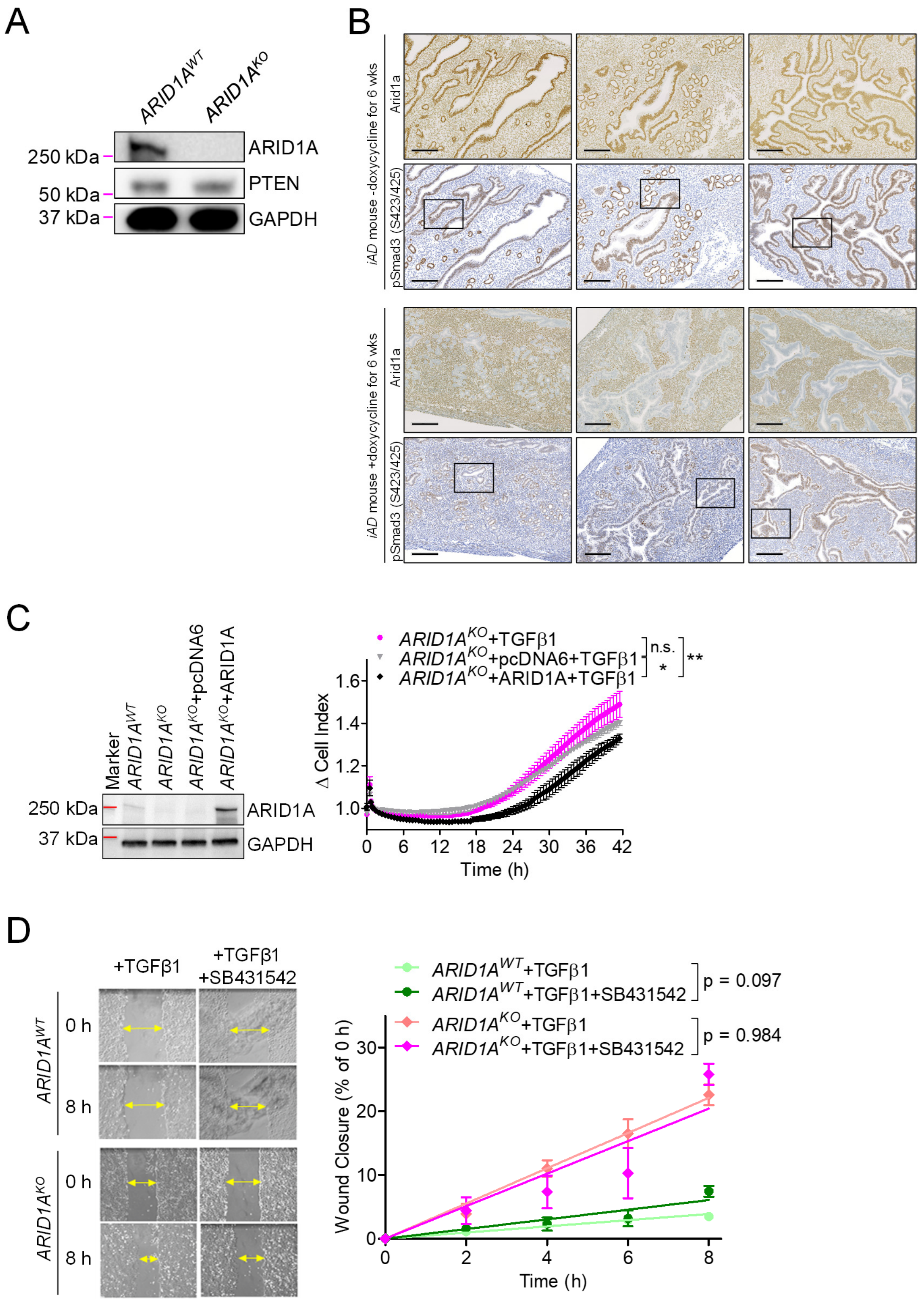
A
ARID1A^WT
ARID1A^KO
250 kDa
50 kDa
37 kDa
ARID1A
PTEN
GAPDH
B
iAD mouse -doxycycline for 6 wks
Arid1a
pSmad3 (S423/425)
iAD mouse +doxycycline for 6 wks
Arid1a
pSmad3 (S423/425)
C
Marker
ARID1A^WT
ARID1A^KO
ARID1A^KO+pcDNA6
ARID1A^KO+ARID1A
250 kDa
37 kDa
ARID1A
GAPDH
ARID1A^KO+TGFβ1
ARID1A^KO+pcDNA6+TGFβ1
ARID1A^KO+ARID1A+TGFβ1
n.s.
*
**
Δ Cell Index
Time (h)
D
+TGFβ1
+TGFβ1 +SB431542
ARID1A^WT
ARID1A^KO
0 h
8 h
ARID1A^WT+TGFβ1
ARID1A^WT+TGFβ1+SB431542
p = 0.097
ARID1A^KO+TGFβ1
ARID1A^KO+TGFβ1+SB431542
p = 0.984
Wound Closure (% of 0 h)
Time (h)

E

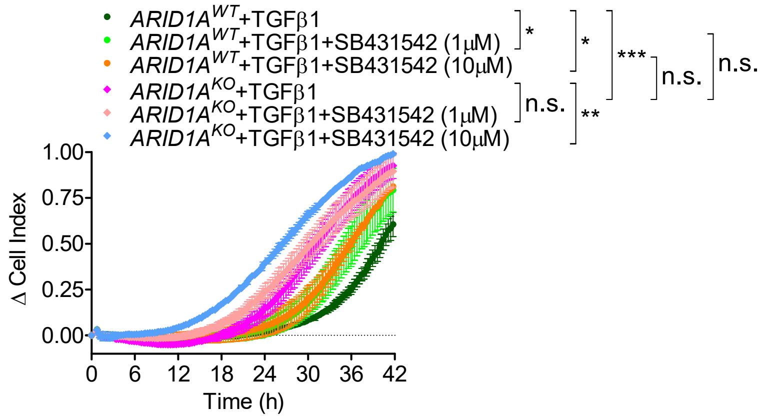

F

Genes in TGF-β signaling pathway that are directly regulated by ARID1A

Gene Name	Log_2 (fold change)	p-value	q value
BMP4	-4.72	5.00E-05	0.0002
DUSP4	-8.47	8.50E-04	0.0030
ID4	5.18	5.00E-05	0.0002
TGFBI	-1.08	5.00E-05	0.0002
TGFBR2	-1.14	5.00E-05	0.0002
THBS1	0.82	1.00E-04	0.0002
TNC	-7.67	5.00E-05	0.0002

Fold change of expression levels in *ARID1A*^{KO}/*ARID1A*^{WT} cells

Supplementary Figure 4. Loss of *ARID1A* attenuates cellular response to inhibitory TGF-β signal.
(A) Immunoblot images showing similar levels of PTEN expression in the isogenic *ARID1A*^{WT} and *ARID1A*^{KO} human endometrial epithelial cell lines. **(B)** Representative Arid1a and pSmad3 (Ser465/467) immunohistochemical stains of *iAD* mouse uterine tissues. Mice were treated with doxycycline for 6 weeks. High magnification images of the insets are shown in Figure 4A. Scale bars, 200 µm. **(C)** Cell invasion capacity measured in *ARID1A*^{KO} cells ectopically re-expressing ARID1A. Immunoblot images showing ARID1A protein re-expressed after a pcDNA6 plasmid encoding full-length ARID1A cDNA was transfected to *ARID1A*^{KO} cells (right panel). pcDNA6 empty vector was used as transfection control. GAPDH was used as a loading control. Cell invasion assays were performed using the xCELLigence RTCA real-time monitoring system. In the presence of exogenous TGF-β1 ligand, re-expression of ARID1A in *ARID1A*^{KO} decreased the ability of cells to invade through the Matrigel layer (black line, right panel) in comparison to the parental *ARID1A*^{KO} cells (magenta) and *ARID1A*^{KO} cells transfected with pcDNA6 empty vector (gray). Data are expressed as mean ± SEM (n = 3). $^{*}p < 0.05$; $^{**}p < 0.01$; n.s., not significant; as determined by one-way ANOVA with Bonferroni's multiple comparison post-test by comparing two groups over time. **(D)** Representative images from a single experiment of the wound healing assay performed on *ARID1A*^{WT} and *ARID1A*^{KO} isogenic cells treated

with TGF-β1 in the absence or presence of SB431542 (left panel). Effects were quantified by the distance of the gaps in the wounds (right panel). Yellow arrows indicate the size of the wound. Light green indicates $ARID1A^{WT}$ treated with TGF-β1, dark green indicates $ARID1A^{WT}$ treated with TGF-β1 and SB431542, orange indicates $ARID1A^{KO}$ treated with TGF-β1, magenta indicates $ARID1A^{KO}$ treated with TGF-β1 and SB431542. Mean±SEM (n=3, from a single experiment) is shown in the quantification graph. Statistical analysis was determined using slope comparison - linear regression test. **(E)** Cell invasion capacity measured in $ARID1A^{WT}$ and $ARID1A^{KO}$ cells treated with 1 μM and 10 μM of TGF-β signaling inhibitor, SB431542. Cell invasion assays were performed using the xCELLigence RTCA real-time monitoring system. SB431542 enhanced the ability of $ARID1A^{WT}$ cells to invade through the Matrigel layer (dark green vs light green and orange lines) to a level that is comparable to $ARID1A^{KO}$ cells (light green and orange vs magenta lines). In comparison, increase invasion capacity of $ARID1A^{KO}$ cells can only be observed at the higher dosage of SB431542 (magenta vs blue lines). Data are expressed as mean ± SEM (n = 3). *p < 0.05; **p < 0.01; ***p < 0.001; n.s., not significant; as determined by one-way ANOVA with Bonferroni's multiple comparison post-test by comparing two groups over time. **(F)** Fold change of expression levels ($ARID1A^{KO}$/$ARID1A^{WT}$ cells) in some of the ARID1A directly targeted genes involving TGF-β pathway.

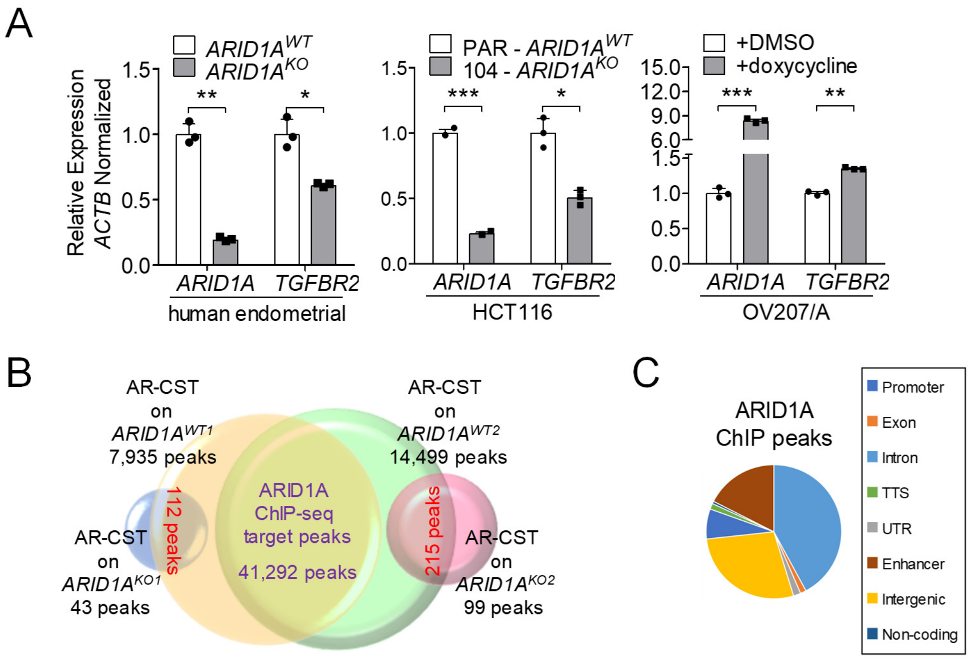

D

HOMER rank	Motifs for ARID1A ChIP-seq target peaks	*p*-value	% of targets	Canonical motifs match
1	ATGAGTCATC	10^{-11988}	46.7%	JUNB
2	ACATTCCT	10^{-1121}	20.0%	TEAD4
3	TTTGTGGTTT	10^{-638}	7.1%	RUNX1
4	AGTCATTTCCTG	10^{-288}	5.6%	EWS:ERG
5	TGGCAGCCTGCC	10^{-248}	10.8%	NF1

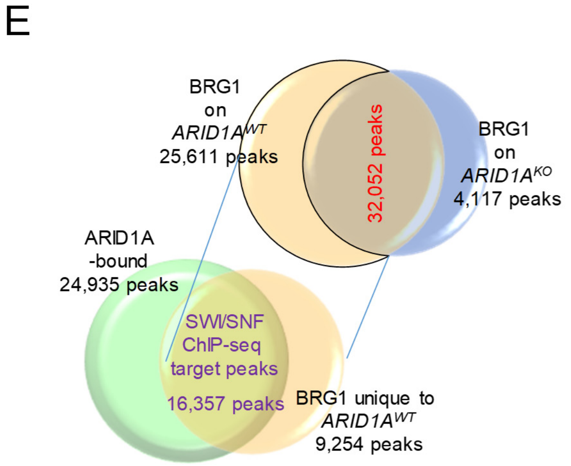

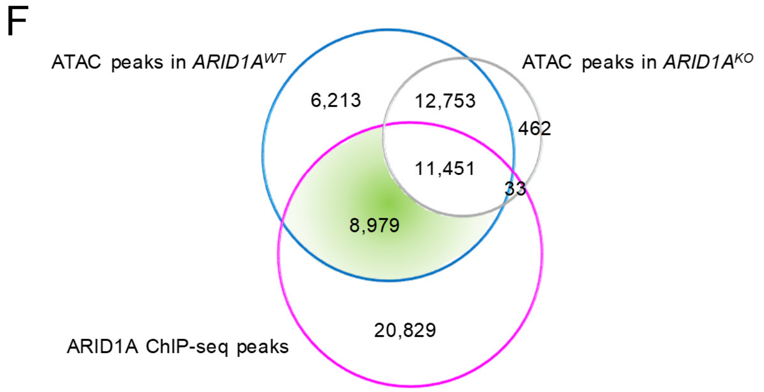

G

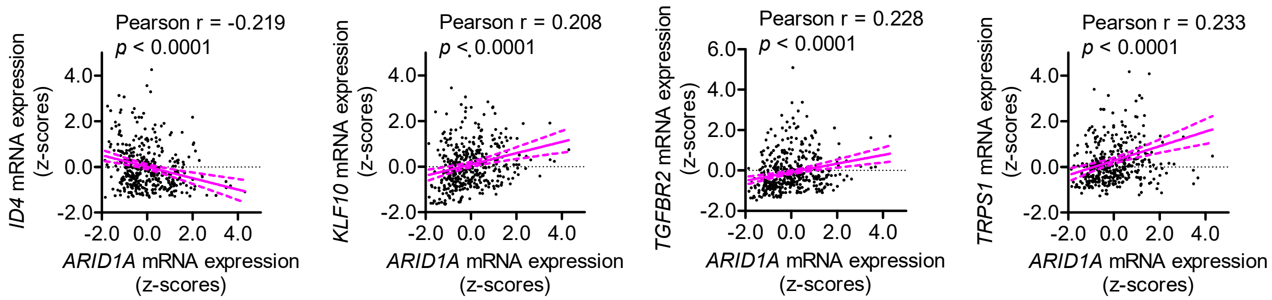

Supplementary Figure 5. Integrated analysis of ChIP-seq and RNA-seq data from isogenic *ARID1A*^{WT} and *ARID1A*^{KO} cells identifies ARID1A directly-regulated transcriptome.
(A) Regulation of *TGFBR2* mRNA expression levels by ARID1A. Quantitative real time PCR demonstrates suppression of *TGFBR2* mRNA expression after *ARID1A* knockout in both isogenic human endometrial epithelial cells and HCT116 human colon cancer cells. Reintroduction of *ARID1A* in OV207 ovarian clear cells leads to up-regulation of *TGFBR2* mRNA expression. *β-actin* (*ACTB*) was used as an internal loading control. Data are expressed as mean ± SD (n = 3). *p < 0.05; **p < 0.01; ***p < 0.001; paired two-tailed Student *t*-test. **(B)** Diagrams showing number of overlapping peaks in ChIP-seq studies performed on *ARID1A*^{KO} and *ARID1A*^{WT} cells. ARID1A ChIP-seq peaks (41,292 peaks) identified by overlapping data from two biological replicates. **(C)** Genomic distribution of ARID1A ChIP-seq target peaks in *ARID1A*^{WT} and *ARID1A*^{KO} cells. The distribution was categorized into promoter, exon, intron, transcription termination site (TTS), untranslated region (UTR), enhancer, intergenic, and non-coding regions. **(D)** The top five transcription factor DNA binding motifs enriched in ARID1A ChIP-seq target peaks. The DNA binding motifs were identified by HOMER Motif Analysis and ranked by *p*-value. **(E)** Diagrams showing BRG1 ChIP-seq peaks specific to *ARID1A*^{WT} cells (25,511 peaks) were overlaid on ARID1A ChIP-seq peaks identified in (B), which results in 16,357 peaks shared between BRG1 and ARID1A ChIPs and is designated as SWI/SNF ChIP target peaks. **(F)** Venn diagrams showing ATAC-seq peaks identified in *ARID1A*^{WT} cells (39,396 peaks, blue circle) and *ARID1A*^{KO} cells (24,699 peaks, grey circle) that were overlaid on ARID1A ChIP-seq peaks identified in (B), which results in 8,979 ARID1A-specific ATAC peaks (green highlight). **(G)** Pearson's correlation of mRNA expression between *ARID1A* and its direct target genes, *ID4*, *KLF10*, *TGFBR2*, and *TRPS1*, in the TCGA uterine endometrial carcinoma PanCancer Atlas dataset. Individual dots represent z-scores data obtained from one patient. The solid magenta lines represent linear regression. Areas between dotted magenta lines: 95% confidence intervals. p-values were determined by Pearson Correlation.

Figure 4B

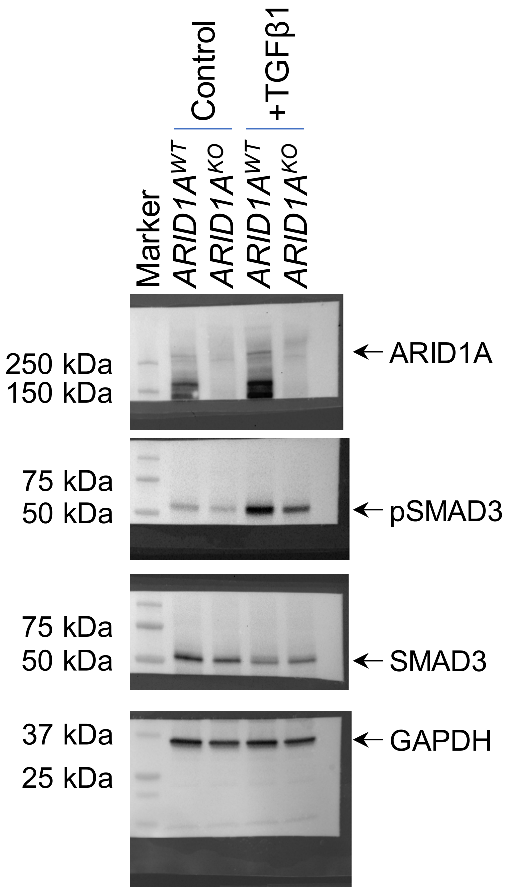

Supplementary Figure 4A

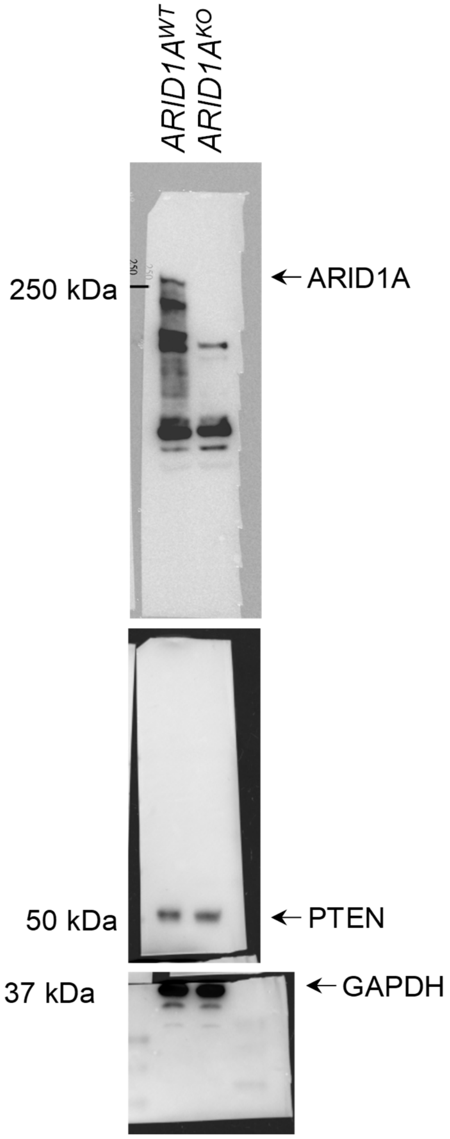

Supplementary Figure 4C

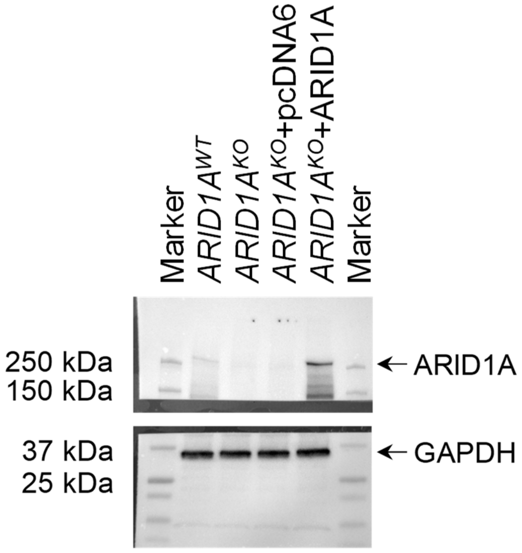

Supplement: Supplementary file 1 — Supplementary Information [file 41467_2020_16416_MOESM1_ESM.pdf]
