# Supplementary material for: Inactivation of Arid1a in the endometrium is associated with endometrioid tumorigenesis through transcriptional reprogramming
Source: Nat Commun. 2020 Jun 1;11:2717. doi: 10.1038/s41467-020-16416-0 (PMC7264300; doi:10.1038/s41467-020-16416-0)
Supplement: Supplementary file 3 — Description of Additional Supplementary Files [file 41467_2020_16416_MOESM3_ESM.pdf]

Description of Additional Supplementary Files

File Name: Supplementary Data 1
Description: Differentially expressed genes between iPD and iPAD uterine tumors.

File Name: Supplementary Data 2
Description: Upstream regulators of Arid1a-associated mouse transcriptome

File Name: Supplementary Data 3
Description: Differentially expressed genes identified in two pairs of isogenic ARID1AWT and ARID1AKO human endometrial epithelial cells.

File Name: Supplementary Data 4
Description: Upstream regulators of ARID1A-associated transcriptome in human endometrial epithelial cells.

File Name: Supplementary Data 5
Description: Gene Ontology enrichment commonly identified in ARID1A-associated transcriptomes of mouse uterine tumors and human endometrial epithelial cells.

File Name: Supplementary Data 6
Description: RNA-seq and ChIP-seq data quality and mapping statistics.

File Name: Supplementary Data 7
Description: ARID1A directly targeted genes identified in two pairs of ARID1AWT and ARID1AKO cells.

File Name: Supplementary Data 8
Description: Genes in the TGF-β signaling pathway that are directly regulated by ARID1A.

File Name: Supplementary Data 9
Description: Ingenuity canonical pathways enriched in ARID1A directly targeted genes.

File Name: Supplementary Data 10
Description: List of primers used in this study
