# Supplementary material for: Inactivation of Arid1a in the endometrium is associated with endometrioid tumorigenesis through transcriptional reprogramming
Source: Nat Commun. 2020 Jun 1;11:2717. doi: 10.1038/s41467-020-16416-0 (PMC7264300; doi:10.1038/s41467-020-16416-0)
Supplement: Supplementary file 14 — Reporting Summary [file 41467_2020_16416_MOESM14_ESM.pdf]

nature research

Corresponding author(s): Shih, Ie-Ming
Wang, Tian-Li
Suryo Rahmanto, Yohan

Last updated by author(s): Apr 18, 2020

Reporting Summary

Nature Research wishes to improve the reproducibility of the work that we publish. This form provides structure for consistency and transparency in reporting. For further information on Nature Research policies, see Authors & Referees and the Editorial Policy Checklist.

Statistics

For all statistical analyses, confirm that the following items are present in the figure legend, table legend, main text, or Methods section.

n/a	Confirmed	
☐	☒	The exact sample size (*n*) for each experimental group/condition, given as a discrete number and unit of measurement
☐	☒	A statement on whether measurements were taken from distinct samples or whether the same sample was measured repeatedly
☐	☒	The statistical test(s) used AND whether they are one- or two-sided<br>*Only common tests should be described solely by name; describe more complex techniques in the Methods section.*
☒	☐	A description of all covariates tested
☐	☒	A description of any assumptions or corrections, such as tests of normality and adjustment for multiple comparisons
☐	☒	A full description of the statistical parameters including central tendency (e.g. means) or other basic estimates (e.g. regression coefficient) AND variation (e.g. standard deviation) or associated estimates of uncertainty (e.g. confidence intervals)
☐	☒	For null hypothesis testing, the test statistic (e.g. *F*, *t*, *r*) with confidence intervals, effect sizes, degrees of freedom and *P* value noted<br>*Give P values as exact values whenever suitable.*
☒	☐	For Bayesian analysis, information on the choice of priors and Markov chain Monte Carlo settings
☒	☐	For hierarchical and complex designs, identification of the appropriate level for tests and full reporting of outcomes
☐	☒	Estimates of effect sizes (e.g. Cohen's *d*, Pearson's *r*), indicating how they were calculated

Our web collection on statistics for biologists contains articles on many of the points above.

Software and code

Policy information about availability of computer code

Data collection	All software used in this study for data collection are either commercially available or open source.<br>RTCA Software Package 1.2<br>SimplePCI automated image capture software<br>Image Lab ver. 6.0
Data analysis	All software used in this study for data analysis are either commercially available or open source.<br>Galaxy, an open access web-based program that contains a variety of next-generation sequencing analysis tools<br>TopHat (ver. 2.1.0)<br>Cufflinks (ver. 2.2.1.0)<br>Cuffmerge (ver. 2.2.1.0)<br>Cuffdiff (ver. 2.2.1.3)<br>Bowtie (ver. 0.12.7)<br>SAMtools (ver. 0.1.19)<br>MACS2 (ver. 2.1.0)<br>HOMER (ver. 4.7.2)<br>deepTools (ver. 2.4.3)<br>CellTracker<br>ImageJ 'Chemotaxis and Migration Tool'<br>GraphPad Prism 5<br>GSEA ver. 4.0.1<br>Image Lab ver. 6.0

For manuscripts utilizing custom algorithms or software that are central to the research but not yet described in published literature, software must be made available to editors/reviewers. We strongly encourage code deposition in a community repository (e.g. GitHub). See the Nature Research guidelines for submitting code & software for further information.

Data

Policy information about availability of data

All manuscripts must include a data availability statement. This statement should provide the following information, where applicable:

- Accession codes, unique identifiers, or web links for publicly available datasets
- A list of figures that have associated raw data
- A description of any restrictions on data availability

The accession numbers for the data for ChIP-seq, ATAC-seq, and RNA-seq reported in this study are NCBI GEO GSE106665.
All reagents and experimental data are available from the corresponding author upon request.

Field-specific reporting

Please select the one below that is the best fit for your research. If you are not sure, read the appropriate sections before making your selection.

☒ Life sciences ☐ Behavioural & social sciences ☐ Ecological, evolutionary & environmental sciences

For a reference copy of the document with all sections, see nature.com/documents/nr-reporting-summary-flat.pdf

Life sciences study design

All studies must disclose on these points even when the disclosure is negative.

Sample size	Sufficient sample sizes were chosen for each experiment to determine whether the outcome was statistically significant.
Data exclusions	No data were excluded from this study.
Replication	We confirmed that all attempts to replicate experiments were successful. All ChIP-seq, ATAC-seq, and RNA-seq experiments data are derived from 2 biological replicates. In each experiments, a minimum of 3 technical replicates were used.
Randomization	iPAD, iPD, and iAD were selected randomly for doxycycline-induced gene knockout in the endometrial epithelium.
Blinding	Blinding was not implemented in this study.

Reporting for specific materials, systems and methods

We require information from authors about some types of materials, experimental systems and methods used in many studies. Here, indicate whether each material, system or method listed is relevant to your study. If you are not sure if a list item applies to your research, read the appropriate section before selecting a response.

Materials & experimental systems

n/a	Involved in the study
☐	☒ Antibodies
☐	☒ Eukaryotic cell lines
☒	☐ Palaeontology
☐	☒ Animals and other organisms
☒	☐ Human research participants
☒	☐ Clinical data

Methods

n/a	Involved in the study
☐	☒ ChIP-seq
☒	☐ Flow cytometry
☒	☐ MRI-based neuroimaging

Antibodies

Antibodies used

Rabbit monoclonal anti-ARID1A (for ChIP-seq and WB) Cell Signaling Technology 12354S
Rabbit monoclonal anti-BRG1 antibody (for ChIP-seq) Abcam ab110641
Rabbit polyclonal anti-RNA polymerase II phospho S2 (for ChIP-seq) Abcam ab5095
Rabbit monoclonal anti-PTEN (for WB and IHC) Cell Signaling Technology 9188S
Rabbit monoclonal anti-Smad3 (for WB) Cell Signaling Technology 9523S
Rabbit monoclonal anti-Smad3 (phospho S423 + S425) (for WB and IHC) Abcam ab52903
Rabbit polyclonal anti-PAX8 (for IHC) Proteintech 10336-1-AP
Rabbit polyclonal anti-ARID1A (for IHC) Sigma-Aldrich HPA005456
Rabbit monoclonal anti-phospho Akt Ser473 (for IHC) Cell Signaling Technologies 4060S
Goat anti-rabbit IgG, HRP-linked Antibody (for Protein-DNA) Cell Signaling Technology 7074S

Validation

All antibodies were validated by the supplier or published previously

Eukaryotic cell lines

Policy information about cell lines

Cell line source(s)	The immortalized parental human endometrial epithelial and the first pair of isogenic ARID1AWT and ARID1AKO cells used in this study have been reported previously by our group (JBC 2016 291:9690-9699). A second isogenic line of human endometrial epithelial cells with ARID1A knockout were generated in the current study. Acquisition of tissue specimens was approved by the Institutional Review Board at the Johns Hopkins Hospital, Baltimore, Maryland.
Authentication	STR authentication were carried out by Johns Hopkins GRCF DNA Services/FAF facility.
Mycoplasma contamination	All cell lines tested negative for mycoplasma by Johns Hopkins GRCF DNA Services/FAF facility.
Commonly misidentified lines (See ICLAC register)	No commonly misidentified cell lines were used.

Animals and other organisms

Policy information about studies involving animals; ARRIVE guidelines recommended for reporting animal research

Laboratory animals	Arid1aflox/flox mice on the 129S1 background were obtained from the Jackson Laboratory. Ptenflox/flox mice on the BALB/c background (Strain C;129S4-Ptentm1Hwu/J) were obtained from the Jackson Laboratory. iPAD, iPD, and iAD mouse models with Arid1a and Pten individual or combined knockout in the uterine epithelium were generated by crossing Pax8-Cre mice (Cancer Cell 2013 24:751-765) with Arid1aflox/flox mice, Ptenflox/flox mice, and Arid1aflox/flox;Ptenflox/flox mice. All experimental mice were maintained on a mixed genetic background (C57BL/6, BALB/c, and S129). Knockout was initiated by treating mice with doxycycline either through oral gavage (2 mg/mouse/day) or subcutaneous implantation of doxycycline pellets (200 mg) when they reached puberty (6-8 weeks old).
Wild animals	Study did not involve wild animals
Field-collected samples	Study did not involve samples collected from the field
Ethics oversight	All of the animal procedures were approved by the Johns Hopkins University Animal Care Committee.

Note that full information on the approval of the study protocol must also be provided in the manuscript.

ChIP-seq

Data deposition

☒ Confirm that both raw and final processed data have been deposited in a public database such as GEO.

☒ Confirm that you have deposited or provided access to graph files (e.g. BED files) for the called peaks.

Data access links *May remain private before publication.*	GSE106665
Files in database submission	GSM3537201 ChIP-Seq: ARID1A-WT1 AR-CST rep1<br>GSM3537202 ChIP-Seq: ARID1A-WT1 AR-CST rep2<br>GSM3537203 ChIP-Seq: ARID1A-WT1 AR-CST rep3<br>GSM3537204 ChIP-Seq: ARID1A-KO1 AR-CST rep1<br>GSM3537205 ChIP-Seq: ARID1A-KO1 AR-CST rep2<br>GSM3537206 ChIP-Seq: ARID1A-KO1 AR-CST rep3<br>GSM3537207 ChIP-Seq: ARID1A-WT2 AR-CST rep1<br>GSM3537208 ChIP-Seq: ARID1A-WT2 AR-CST rep2<br>GSM3537209 ChIP-Seq: ARID1A-WT2 AR-CST rep3<br>GSM3537210 ChIP-Seq: ARID1A-KO2 AR-CST rep1<br>GSM3537211 ChIP-Seq: ARID1A-KO2 AR-CST rep2<br>GSM3537212 ChIP-Seq: ARID1A-KO2 AR-CST rep3<br>GSM3537213 ChIP-Seq: ARID1A-WT1 BRG1 rep1<br>GSM3537214 ChIP-Seq: ARID1A-WT1 BRG1 rep2<br>GSM3537215 ChIP-Seq: ARID1A-WT1 BRG1 rep3<br>GSM3537216 ChIP-Seq: ARID1A-KO1 BRG1 rep1<br>GSM3537217 ChIP-Seq: ARID1A-KO1 BRG1 rep2<br>GSM3537218 ChIP-Seq: ARID1A-KO1 BRG1 rep3<br>GSM3537219 ChIP-Seq: ARID1A-WT2 BRG1 rep1<br>GSM3537220 ChIP-Seq: ARID1A-WT2 BRG1 rep2<br>GSM3537221 ChIP-Seq: ARID1A-WT2 BRG1 rep3<br>GSM3537222 ChIP-Seq: ARID1A-KO2 BRG1 rep1<br>GSM3537223 ChIP-Seq: ARID1A-KO2 BRG1 rep2<br>GSM3537224 ChIP-Seq: ARID1A-KO2 BRG1 rep3<br>GSM3537225 ChIP-Seq: ARID1A-WT1 RNA Pol2 rep1

GSM3537226 ChIP-Seq: ARID1A-WT1 RNA Pol2 rep2
GSM3537227 ChIP-Seq: ARID1A-WT1 RNA Pol2 rep3
GSM3537228 ChIP-Seq: ARID1A-KO1 RNA Pol2 rep1
GSM3537229 ChIP-Seq: ARID1A-KO1 RNA Pol2 rep2
GSM3537230 ChIP-Seq: ARID1A-KO1 RNA Pol2 rep3
GSM3537231 ChIP-Seq: ARID1A-WT2 RNA Pol2 rep1
GSM3537232 ChIP-Seq: ARID1A-WT2 RNA Pol2 rep2
GSM3537233 ChIP-Seq: ARID1A-WT2 RNA Pol2 rep3
GSM3537234 ChIP-Seq: ARID1A-KO2 RNA Pol2 rep1
GSM3537235 ChIP-Seq: ARID1A-KO2 RNA Pol2 rep2
GSM3537236 ChIP-Seq: ARID1A-KO2 RNA Pol2 rep3

Genome browser session (e.g. UCSC)

N/A

Methodology

Replicates

2 biological replicates with 3 technical replicates each

Sequencing depth

NextSeq500 platform with single-end reads of 75 bases. ChIP-seq data quality and mapping statistics are shown in Supplementary Table 5.

Antibodies

Rabbit monoclonal anti-ARID1A (for ChIP-seq) Cell Signaling Technology 12354S
Rabbit monoclonal anti-BRG1 antibody (for ChIP-seq) Abcam ab110641
Rabbit polyclonal anti-RNA polymerase II phospho S2 (for ChIP-seq) Abcam ab5095

Peak calling parameters

ChIP-seq peaks were called by MACS2 v2.1.040 using matched sample and input BAM files. For each transcription factor (ARID1A, BRG1, and RNA Pol II), narrow peaks were called by setting the q-value at 0.01.

Data quality

ChIP-seq data quality and mapping statistics are shown in Supplementary Table 5.

Software

Bowtie (ver. 0.12.7)
SAMtools (ver. 0.1.19)
MACS2 (ver. 2.1.0)
HOMER (ver. 4.7.2)
deepTools (ver. 2.4.3)
